# World's first live AI‐assisted augmented reality (AR) robotic colon surgery with instrument deocclusion: Navigating the future of surgery

**DOI:** 10.1111/codi.70296

**Published:** 2025-11-10

**Authors:** Thalia Petropoulou, Jente Simoens, Andreas Polydorou, Pieter De Backer, Alex Mottrie

**Affiliations:** ^1^ Second Department of Surgery, Aretaieion University Hospital National and Kapodistrian University of Athens Athens Greece; ^2^ Department of Robotic Surgery The Athens Euroclinic Group Athens Greece; ^3^ ORSI Academy Ghent Belgium

**Keywords:** artificial intelligence, augmented reality, colorectal surgery, intraoperative guidance, minimally invasive surgery, right hemicolectomy, robotic surgery, surgical innovation

## BACKGROUND

Artificial intelligence (AI) and augmented reality (AR) are transforming the landscape of minimally invasive surgery. We present the world's first live integration of an AI‐assisted AR overlay with real‐time instrument deocclusion in robotic colorectal surgery.

## METHODS

A real surgical case of robotic right colectomy was performed using a da Vinci Xi platform enhanced by an AI‐driven AR system. The technology generated three‐dimensional anatomical overlays while simultaneously applying instrument deocclusion algorithms to maintain continuous visualization of critical anatomy (Video 1).

**VIDEO 1:** The video demonstrates the integration of an AI algorithm within the robotic platform, providing real‐time augmented visualization and de‐occlusion of surgical instruments during key operative steps. This technology was used in a non‐interventional, observational capacity and did not alter the surgical technique or treatment pathway. Video content can be viewed at https://onlinelibrary.wiley.com/doi/10.1111/codi.70296.

## RESULTS

The procedure was successfully completed without conversion, demonstrating the technical feasibility of combining AI‐based overlays and deocclusion in a complex intraoperative environment. The system allowed stable AR guidance despite dynamic camera movements and instrument interference. From the operating surgeon's perspective, the AR overlay was useful, non‐disruptive to workflow, and enhanced anatomical orientation. These impressions are preliminary and require validation in prospective studies.

## CONCLUSION

This proof‐of‐concept highlights the feasibility of AI‐assisted AR with instrument deocclusion in robotic colorectal surgery and represents an important step toward digital surgery of the future. While no formal outcomes or structured user assessments were collected in this initial demonstration, future studies will focus on systematic evaluation of usability, surgeon feedback, and clinical performance metrics.

## AUTHOR CONTRIBUTIONS

**Thalia Petropoulou:** Conceptualization; investigation; writing – original draft; methodology; validation; visualization; writing – review and editing; formal analysis. **Jente Simoens:** Methodology; validation; software. **Andreas Polydorou:** Investigation; validation; supervision. **Pieter De Backer:** Conceptualization; methodology; validation; software; formal analysis; data curation. **Alex Mottrie:** Methodology; validation; software; supervision.

## FUNDING INFORMATION

No funding was used.

## CONFLICT OF INTEREST STATEMENT

No conflict of interest between the authors.

## ETHICS STATEMENT

This study was conducted in accordance with the ethical standards of the 1964 Helsinki Declaration and its later amendments.

## Data Availability

The data that support the findings of this study are available from the corresponding author upon reasonable request.

